# Modified Nanopillar Arrays for Highly Stable and Efficient Photoelectrochemical Water Splitting

**DOI:** 10.1002/gch2.201800027

**Published:** 2018-11-19

**Authors:** Lanyan Huang, Qingguo Meng, Chaoqun Shang, Mingliang Jin, Lingling Shui, Yongguang Zhang, Zhang Zhang, Zhihong Chen, Mingzhe Yuan, Xin Wang, Krzysztof Kempa, Guofu Zhou

**Affiliations:** ^1^ Guangdong Provincial Key Laboratory of Optical Information Materials and Technology & Institute of Electronic Paper Displays South China Academy of Advanced Optoelectronics South China Normal University Guangdong China; ^2^ Shenyang Institute of Automation, Guangzhou Chinese Academy of Sciences Guangzhou Guangdong China; ^3^ International Academy of Optoelectronics at Zhaoqing South China Normal University Guangdong China; ^4^ Department of Physics Boston College Chestnut Hill MA 02467 USA

**Keywords:** graphitic carbon nitride, hydrogen evolution, photoelectrochemistry, quantum dots, TiO_2_ nanopillars

## Abstract

Atomically modified graphitic carbon nitride quantum dots (QDs), characterized by strongly increased reactivity and stability, are developed. These are deposited on arrays of TiO_2_ nanopillars used as a photoanode for the photoelectrochemical water splitting. This photoanode shows excellent stability, with 111 h of continuous work without any performance loss, which outperforms the best‐reported results by a factor of 10. Remarkably, our photoanode produces hydrogen even at zero bias. The excellent performance is attributed to the enhancement of photoabsorption, as well as to the promotion of charge separation between TiO_2_ nanopillars and the QDs.

Solar‐driven water splitting, which extracts hydrogen fuel directly from water, provides a practical and environmentally safe solution to the global energy problem.^1^ Since the early demonstration in 1972,^2^ TiO_2_‐based photoelectrodes have been extensively explored in the photoelectrochemical (PEC) hydrogen generation, due to their low cost, environmental friendliness, and excellent chemical stability.^3^ However, TiO_2_ with its large bandgap can only be excited by ultraviolet irradiation, which constitutes only <5% of the whole solar spectrum. In addition, TiO_2_ suffers from fast carrier recombination.^4^ Intensive research efforts have focused on remedying these drawbacks. The overall photocatalytic efficiency can be enhanced by doping.^5^ To broaden the absorption spectrum, a dye or quantum dot (QD) sensitization was employed,^6^ as well as multijunction hybrid designs with narrow bandgap semiconductors.^7^ In particular, semiconductor QDs have attracted great attention due to their unique optical and electronic properties. It has been reported that TiO_2_ nanostructures, sensitized with QD such as (CdS,^8^ CdTe,^9^ CdSe,^10^ PbS,^11^ etc.) provided substantially enhanced photocurrent. However, serious photocorrosion and considerable potential health and environmental problems due to heavy metals typically used in QD hinder their wide application. The search for an alternative semiconductor led to graphitic carbon nitride (g‐C_3_N_4_), known as a polymeric semiconductor photocatalyst with a bandgap of 2.7 eV.^12^ QDs made of g‐C_3_N_4_ have been developed in recent years.^13^ These QDs are metal free and nontoxic, and have unique fluorescent and stable properties. Zhou and co‐workers^14^ synthesized highly fluorescent g‐C_3_N_4_ QD by solid‐phase method, using urea and sodium citrate materials and subsequent dialysis process. In another approach, Yu and co‐workers^15^ proposed g‐C_3_N_4_‐based heterojunctions for more‐efficient charge separation and transport.

Recently, An and co‐workers^16^ developed a facile method to synthesize g‐C_3_N_4_ QD (CNQD) on a TiO_2_ nanowires composite, demonstrating a significant improvement of PEC performance, as compared with the bare TiO_2_. In this work, we developed an atomic modification procedure for such a composite, which led to further dramatic improvement of PEC performance. Our photoanodes exhibit superior photocurrent density and hydrogen evolution rate (HER), significantly higher than that obtained with other systems. More importantly, our electrodes demonstrate outstanding photocatalytic stability, with more than 111 h of unaffected operation under continuous illumination.

The first step in preparation of our composites is to grow TiO_2_ nanopillar arrays on a transparent, fluorine‐doped tin oxide (FTO) substrate, by employing the commonly used solvothermal method (more details in the Supporting Information).^17^

The modified g‐C_3_N_4_ QDs were synthesized on TiO_2_ nanopillars by using the one‐pot quasi‐chemical vapor deposition (CVD) method^18^ (more details in the Supporting Information). While in the processing in a crucible system^18^ the amount of dicyandiamide (DCD) was fixed (7 g), four different amounts of barbituric acid (BA) were used: 0, 0.15, 0.3, 0.5 g. This led to the corresponding four different composites called in our simplified notation as follows: CNQD@TiO_2_, CNB_0.15_QD@TiO_2_, CNB_0.3_QD@TiO_2_, and CNB_0.5_QD@TiO_2_, respectively.

The electron microscopic study of the CNQD@TiO_2_ is shown in **Figure** **1**. The scanning electron microscopy (SEM) images are shown a) and b), and demonstrate that our TiO_2_ arrays consist of highly dense and vertically aligned nanopillars, which provides a large specific surface area for QD adhesion. The nanopillars have cross‐sectional dimensions ranging from 100 to 200 nm, and lengths of ≈3 µm. There is no significant morphology difference between modified CNQD@TiO_2_ and pristine TiO_2_ (see Figure S1, Supporting Information). The transmission electron microscopy (TEM) image shown in Figure 1c reveals that the surface of TiO_2_ nanopillars is uniformly decorated with QD, each with diameter of 3–6 nm. The high‐resolution TEM (HRTEM) image is shown in Figure 1d, revealing further nanoscopic details of the structure. The inset in this figure shows further magnification (atomic resolution) image of the TiO_2_ nanopillar fragment (marked with a square), which shows that the interatomic lattice spacing is 0.250 nm, which corresponds to the (101) planes of a rutile TiO_2_ structure.

**Figure 1:**
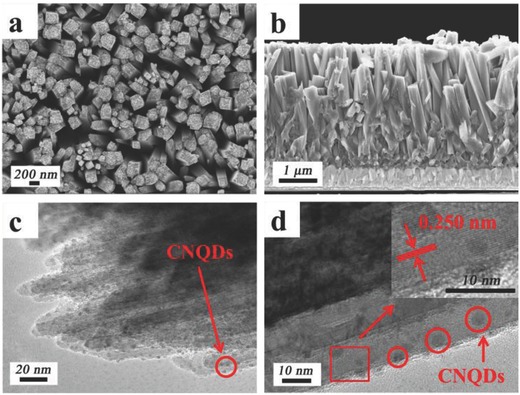
QD decorated TiO_2_ composite (CNQD@TiO_2_). a) Top view and b) side‐view SEM images of the pillar arrays. c) TEM and d) HRTEM images of individual pillars.

The photoluminescence (PL) property of modified CNQDs is also characterized and investigated in this work (see Figure S2, Supporting Information). Several modified CNQDs@TiO_2_ substrates are immersed in water, and the transparent solution of CNQDs is obtained after sonication for 20 min and filter process. The modified CNQDs show excitation‐dependent PL spectra, and the broad peak can be attributed to the abundant N defects in CNQDs, which is similar to the previous report.^19^

**Figure** **2**a shows the X‐ray diffraction (XRD) patterns from all samples FTO, TiO_2_ nanopillars, and the four modified composites. In addition to the eight diffraction peaks of FTO, there are three characteristic peaks from TiO_2_, indexed to typical crystal planes. The lack of obvious diffraction peaks from g‐C_3_N_4_ is due to insufficient volume and poor crystallinity of the CNQD. The X‐ray photoelectron spectroscopy (XPS) signal for C (1s) is shown in Figure 2b, and for N (1s) in c) for modified CNQD@TiO_2_ sample. It demonstrates that the modified composites are composed of C, N, Ti, and O elements (see also Figure S3, Supporting Information). Specifically, a high‐resolution XPS spectrum of C (1s) in Figure 2b shows that the peak centered at 284.8 eV is exclusively attributed to the accidental contamination with the carbon from XPS instrument. The peak at 286.1 eV is assigned to C—O bonds resulted from the pyrolysis of precursors, and the one at 288.0 eV is from carbon atom in the N—C=N group. The XPS spectrum of the N (1s) shown in Figure 2c region can be fitted with three peaks centered at 398.6, 399.7, and 401.2 eV. These are ascribed to the sp^2^‐hybridized nitrogen (C—N=C), nitrogen in tertiary N—C_3_ groups, and amino functional groups with a hydrogen atom (C—N—H), respectively. Compared with pristine TiO_2_, the Ti 2p spectrum of modified CNQDs@TiO_2_ sample shows a 0.4 eV shift to lower binding energy (see Figure S4, Supporting Information). Those results confirm the presence of g‐C_3_N_4_, and the robust interaction between TiO_2_ and CNQDs.

**Figure 2:**
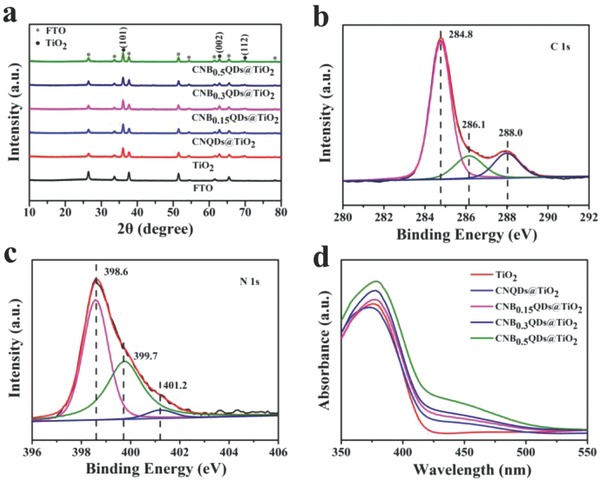
a) XRD patterns of FTO, TiO_2_ nanopillars, and modified composites. High‐resolution XPS spectra of C (1s) b) and N (1s) c). d) The diffuse absorbance spectra in the visible and UV range for all samples. The universal color code (see legend) identifies the samples.

The UV–vis diffuse absorbance spectra displayed in Figure 2d show that the absorption of modified CNBxQDs@TiO_2_ gradually shifts toward longer wavelength with the increasing BA contents. As compared with pristine TiO_2_, CNB_0.5_ QDs@TiO_2_ shows a broadest absorption edge of about 440 nm. That can be ascribed to BA introduction of carbon atoms into the melon‐based carbon nitride structures during copolymerization process, which changes the electronic structure of g‐C_3_N_4_ and thus extends its optical absorption range. Obviously, the combination of TiO_2_ and modified CNQDs contributes to the enhancement of the visible absorption.

The samples have been used as photoanodes in PEC measurements, which is performed in a three‐electrode electrochemical system, with 0.5 m Na_2_SO_4_ electrolyte (pH = 7.62) under simulated solar light illumination at 100 mW cm^−2^. The linear sweep voltammograms displayed in **Figure** **3**a clearly show that both CNBxQD@TiO_2_ and CNQD@TiO_2_ samples show higher photocurrent values and negative shift of the onset potential for water splitting than the pristine TiO_2_ does.

**Figure 3:**
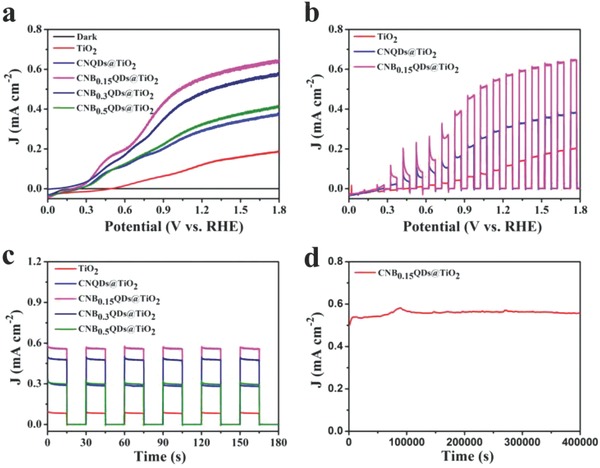
a) The linear sweep voltammograms of TiO_2_, CNQDs@TiO_2_, and modified CNBxQDs@TiO_2_ composite (*x* = 0.15, 0.3, 0.5). b) The linear sweep voltammograms of the pristine TiO_2_, CNQDs@TiO_2_, and modified CNB_0.15_QDs@TiO_2_ composites under intermittent illumination, at a scan rate of 10 mV·s^−1^. c) Chronoamperometry (*J* vs *t*) curves with chopped light illumination at the potential of 0.5 V (vs Ag/AgCl). d) *J* vs *t* curves for CNB_0.15_QDs@TiO_2_ sample at 0.5 V (vs Ag/AgCl) up to 400 000 s (≈111 h), under continuous simulated sunlight illumination at 100 mW cm^−2^.

CNB_1.5_QDs@TiO_2_ (with the content of 1.5 g BA) shows the highest photocurrent density (*J*), and demonstrates only slight enhancement as compared with CNB_0.1_QDs@TiO_2_ (see Figure S5, Supporting Information). The photocurrent density gradually decreases with an increase of the BA content, even though the corresponding absorption in the visible increases. This can be attributed to the formation of defects in the melon‐based carbon nitride structures during the substitution of N in the copolymerization process. The resulting defects facilitate charge separation.

The linear sweep voltammetry measurement performed under intermittent illumination (Figure 3b) shows that the CNQDs@TiO_2_ composite shows remarkable enhancement of photocurrent density as compared with pure TiO_2_. After copolymerization with BA, the optimal sample (CNB_0.15_QDs@TiO_2_) achieves high photocurrent density of 0.57 mA·cm^−2^ at 1.23 V (versus reversible hydrogen electrode (vs RHE)), which is 4.75 times higher than that for the pristine TiO_2_ (0.12 mA·cm^−2^) and 1.9 times higher than CNQDs@TiO_2_ (0.3 mA·cm^−2^), at identical conditions. These results not only demonstrate great advantages of the extended absorption range (into the visible range) and better charge separation, but also suggest that the small size and good dispersion of CNQD on the TiO_2_ nanopillars induce abundant active sites, which enhance the PEC performance.

Figure 3c displays the chronoamperometry curves of samples at 1.15 V versus RHE under discontinuous (chopped) light illumination. The samples show high stability of the photoresponse. More importantly, as shown in Figure 3d, the optimized photoelectrode (CNB_0.15_QDs@TiO_2_) demonstrates excellent stability with only 0.72% decay of photocurrent density (from 0.56 to 0.556 mA·cm^−2^), under continuous illumination of a simulated solar light at 1.15 V versus RHE for more than ≈111 h. This electrode retains the remarkable stability even at 0.65 V versus RHE (Figure S6, Supporting Information) and also retains a relatively stable photocurrent value of 0.065 mA·cm^−2^ after irradiation for 72 000 s (12 h). We attribute this outstanding performance to a strongly reduced photocorrosion by the CNQD decorating the TiO_2_ nanopillars.

The photocatalytic hydrogen generation ability of the samples was also investigated (see experimental details in the Supporting information). As shown in **Figure** **4**, the pristine TiO_2_ exhibits the HER of only of 0.055 µmol·h^−1^·cm^−2^. A significant enhancement of HER up to 0.225 µmol·h^−1^·cm^−2^ occurs already for the CNQD@TiO_2_ sample, but a really dramatic increase, up to 0.8525 µmol·h^−1^·cm^−2^ is achieved in the sample of the optimal composite (CNB_0.15_QDs@TiO_2_).

**Figure 4:**
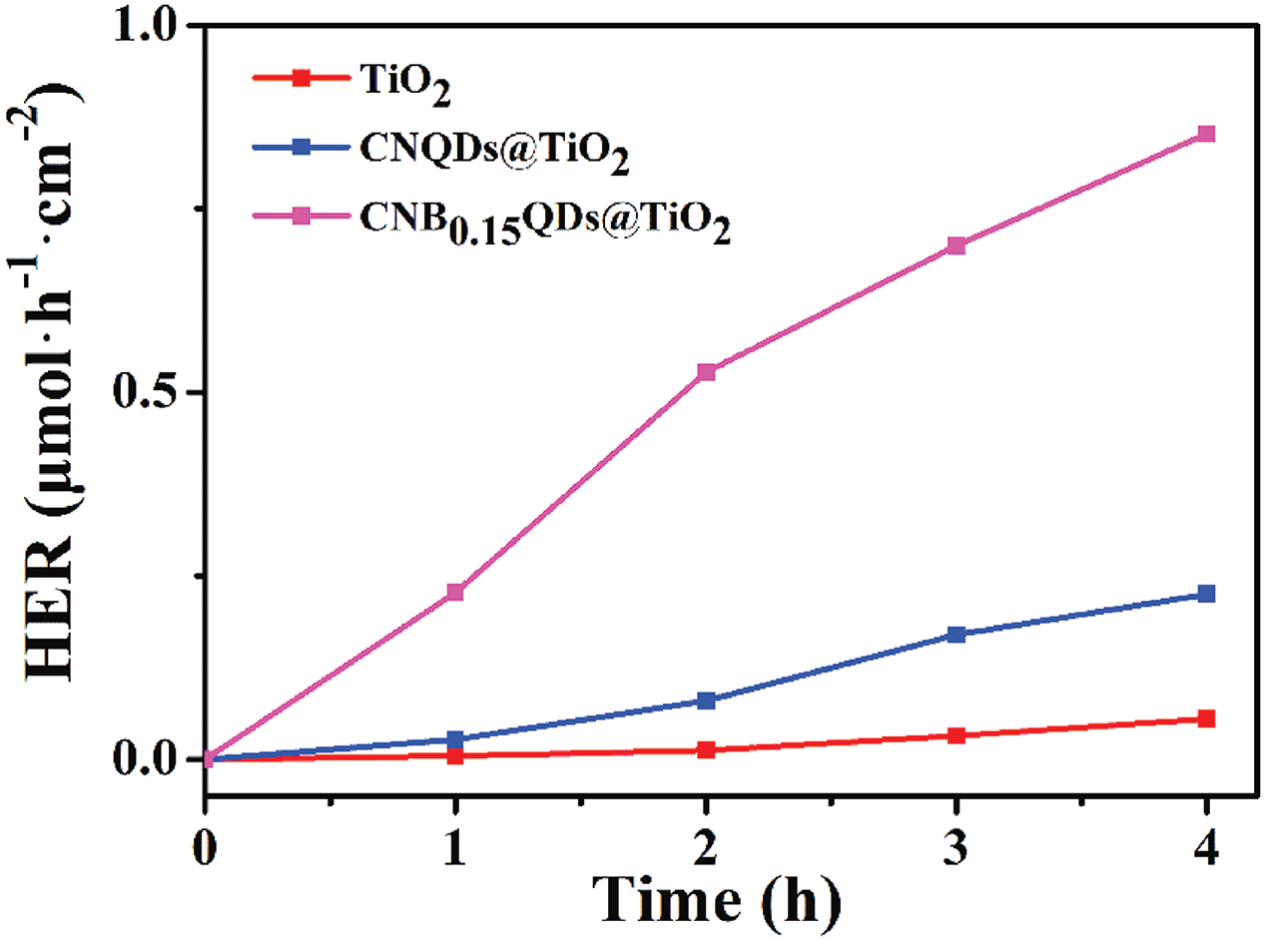
Photocatalytic H_2_ evolution rate of pristine TiO_2_, CNQDs@TiO_2_, and modified CNB_0.15_QDs@TiO_2_ composite in aqueous methanol solution under simulated solar light.

Based on the above results, we provide the following, microscopic explanation of the excellent performance, which we attribute to the atomic level change in the g‐C_3_N_4_ QDs. This is shown schematically in **Figure** **5**. The exposure to dicyandiamide and BA at 550 °C during our process leads to the substitution of N atoms, shown in blue color in the top‐right inset in Figure 5, with C atoms (N‐defects), shown in green color in both insets. The N‐defect sites are more reactive, which improves the PEC performance, and in consistent with the previous reports of induction defects in photocatalytic system.^20^

**Figure 5:**
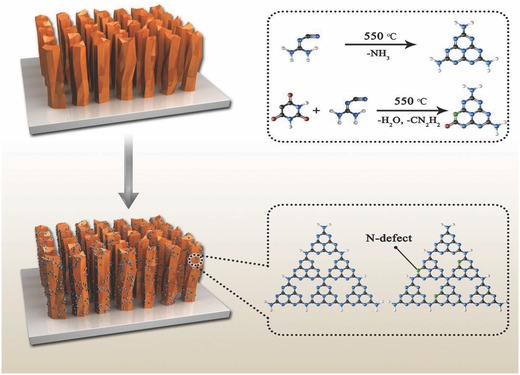
Schematic of the TiO_2_ pillars structures bare (top‐left panel) and QD‐coated (bottom‐left panel). The schematic of the microscopic structural modification of the QDs is shown in the right panels, showing mechanism of formation of N‐defects in the QD atomic lattice: blue atoms (N), black atoms (C), green atoms (C, new formation of N‐defects).

In conclusion, we developed atomically modified graphitic carbon nitride QDs, characterized by strongly increased reactivity and stability. These have been deposited on arrays of TiO_2_ nanopillars, forming composites used as a photoanode for the PEC water splitting. We demonstrate that these photoanodes are highly stable and are characterized by a highly efficient PEC performance. The photoanode based on the best (optimized) composite (CNB_0.15_QDs@TiO_2_) exhibits the highest photocurrent density of 0.57 mA·cm^−2^ at 1.23 V, and an excellent photocatalytic stability of ≈111 h, under continuous illumination. It also demonstrates remarkable hydrogen production, with a rate of 0.8525 µmol·h^−1^·cm^−2^, which is 15.5 times higher than that of a pristine TiO_2_. Thus, this nanocomposite is an excellent candidate for the water‐splitting application.

## Conflict of Interest

The authors declare no conflict of interest.

## Supporting information

Supplementary
