# Supplementary material for: Modified Nanopillar Arrays for Highly Stable and Efficient Photoelectrochemical Water Splitting
Source: Glob Chall. 2018 Nov 19;3(3):1800027. doi: 10.1002/gch2.201800027 (PMC6436580; doi:10.1002/gch2.201800027)
Supplement: Supplementary file 1 — Supplementary [file GCH2-3-1800027-s001.pdf]

Copyright WILEY-VCH Verlag GmbH & Co. KGaA, 69469 Weinheim, Germany, 2018.

Supporting Information

for *Global Challenges,* DOI: 10.1002/gch2.201800027

Modified Nanopillar Arrays for Highly Stable and Efficient Photoelectrochemical Water Splitting

Lanyan Huang, Qingguo Meng, Chaoqun Shang, Mingliang Jin, Lingling Shui, Yongguang Zhang, Zhang Zhang, Zhihong Chen, Mingzhe Yuan, Xin Wang,* Krzysztof Kempa, and Guofu Zhou*

Supporting Information
©Wiley-VCH 2018
69451 Weinheim, Germany

Modified Nanopillar Arrays for Highly Stable and Efficient Photoelectrochemical Water Splitting

Lanyan Huang[1], Qingguo Meng[2], Chaoqun Shang[1], Mingliang Jin[1,4], Lingling Shui[1], Yongguang Zhang[4], Zhang Zhang[4], Zhihong Chen[2,4], Mingzhe Yuan[2], Xin Wang[1,4*], Krzysztof Kempa[3] and Guofu Zhou[1,4]*

Experimental Section

Synthesis of TiO_2 nanopillar arrays. A commonly solvothermal method was carried out to grow TiO_2 nanopillar arrays on transparent fluorinedoped tin oxide (FTO) substrate firstly. In detail, FTO (2*5 cm) was placed into a sealed Teflon reactor (50 mL) containing 18 ml of hydrochloric acid (37 wt %), 0.6 ml of tetrabutyl titanate, and 18 ml of deionized water. The TiO_2 nanopillar arrays on FTO were obtained after the autoclave was kept in an oven at 150 °C for 18 h.

Synthesis of modified CNQDs@TiO_2 composite. The modified g-C_3N_4 QDs were synthesized on TiO_2 nanopillars via a one-pot quasi-CVD method. With the mixed 7 g of DCD and different amounts of BA (0 g, 0.15 g, 0.3 g, 0.5 g) in a crucible (100 ml), a piece of TiO_2 nanopillars substrate(2*5 cm^2) was placed onto the precursors. The crucible system was calcined in a muffle furnace at 550 °C for 3 h before cooling down. The heating rate should be controlled at 2.5 °C/min. Finally, the excess bulk g-C_3N_4 on the surface of the substrate was removed by a stream of nitrogen. And TiO_2 nanopillars sample were treated in the crucible system without any precursor.

Photoelectrochemical measurement: All the PEC measurements were performed in a conventional three-electrode electrochemical system using Pt plate(1 cm^2) as counter electrode and Ag/AgCl electrode (3 M KCl) as reference electrode in 0.5 M Na_2SO_4 electrolyte (pH = 7.62) under simulated solar light illumination at 100 mW/cm^2. A 300 W Xe lamp with AM1.5 filters was choosen as light source. The area of working electrode was controlled at $1cm^2$.

Photocatalytic hydrogen generation Test: Photocatalytic hydrogen generation was carried out in a home-made Pyrex top-irradiation reaction vessel connected to a glass closed gas circulation system. The experiment was performed with a piece of sample (2*4 cm^2) in a 50 mL mixed solution of methanol and water (V/V=1:4) containing 0.375 mg Pt of $H_2PtCl_6 \cdot 6H_2O$ aqueous solution. The reactant solution was evacuated several times to remove air completely prior to irradiation. A cooling water system was used to maintain a constant temperature during the irradiation. A 300 W xenon lamp with AM1.5 filters was chosen as light source, and 100 μL of generated gas was collected intermittently and analyzed by gas chromatography.

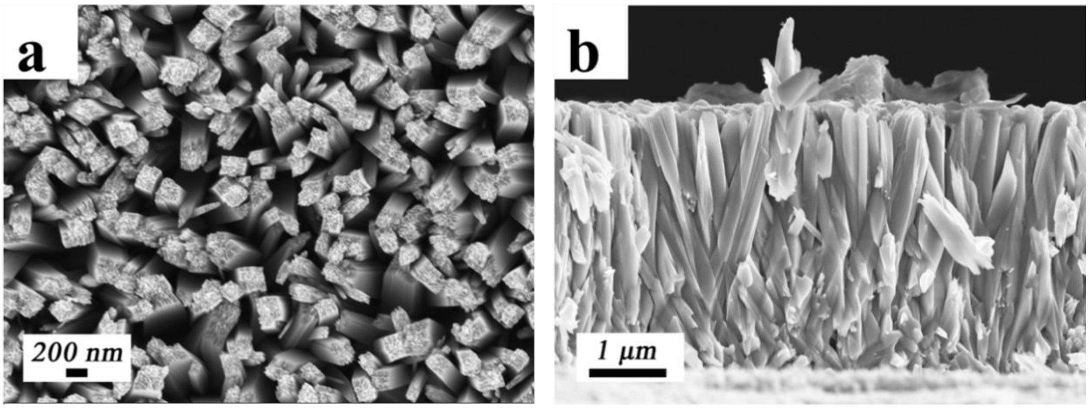

Figure S1. (a) Top view and (b) cross view SEM images of pristine TiO_2 nanostructure.

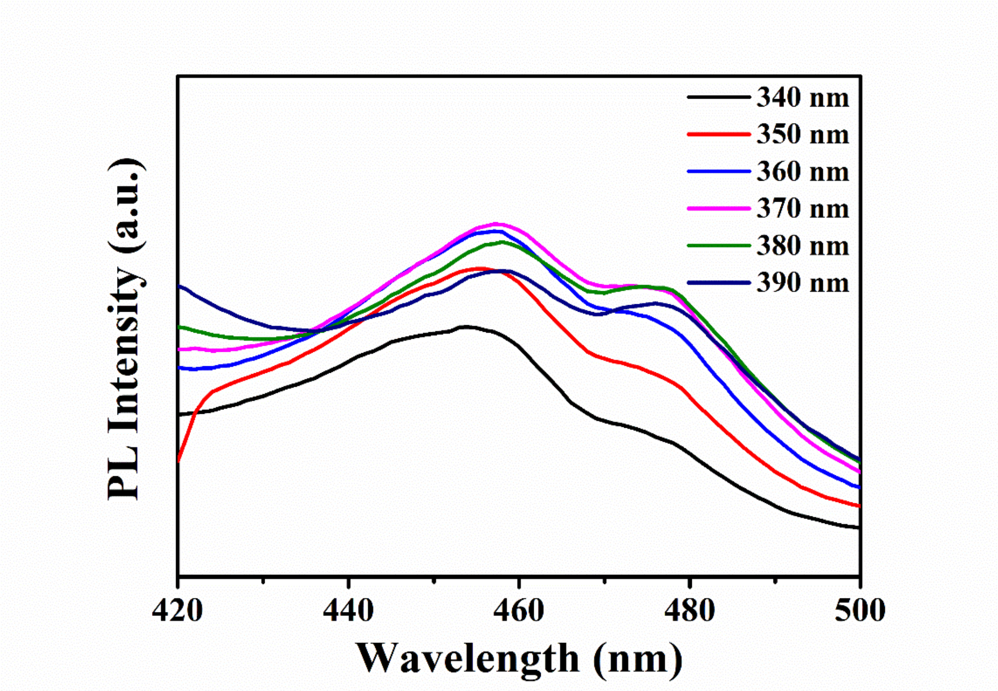

Figure S2. Photoluminescence spectra of modified CNQDs excited under different wavelengths from 340 nm to 390 nm.

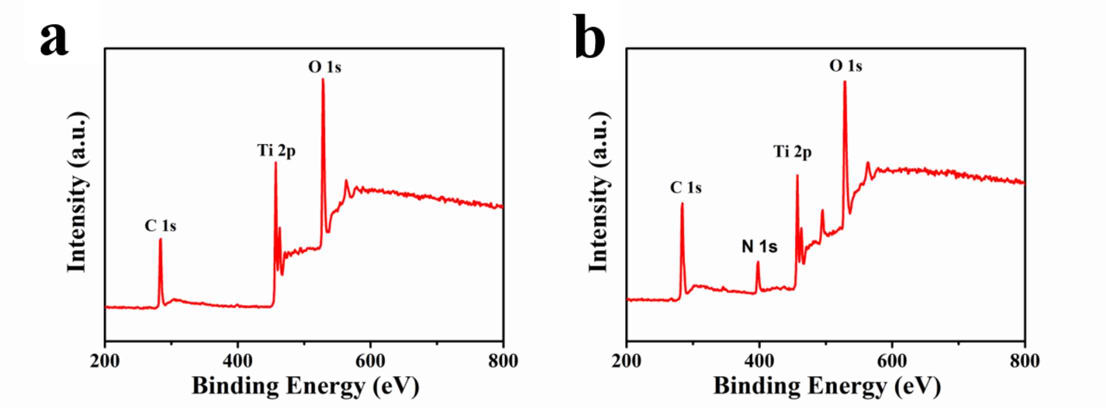

Figure S3. The XPS survey spectrum of (a) pristine TiO_2 and (b) modified CNQDs @ TiO_2 sample.

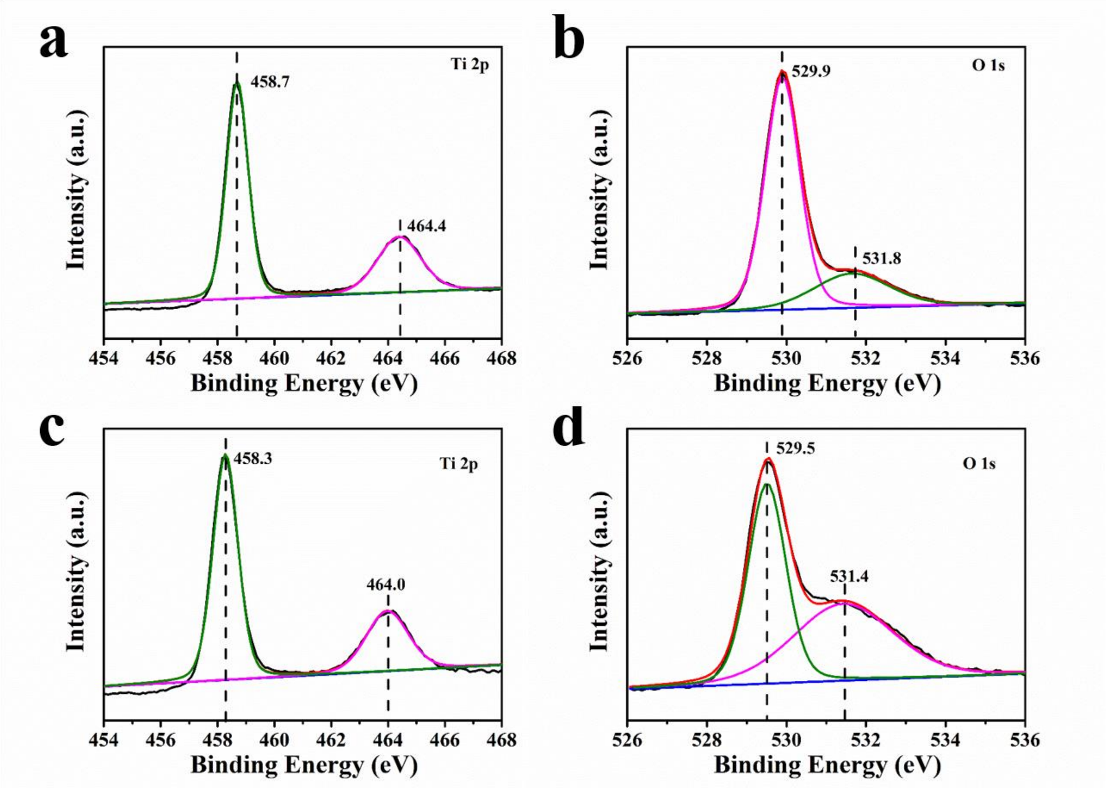

Figure S4. High resolution XPS spectra of (a) Ti 2p and (b) O 1s of pristine TiO_2; (c) Ti 2p and (d) O 1s of modified CNQDs @ TiO_2 sample.

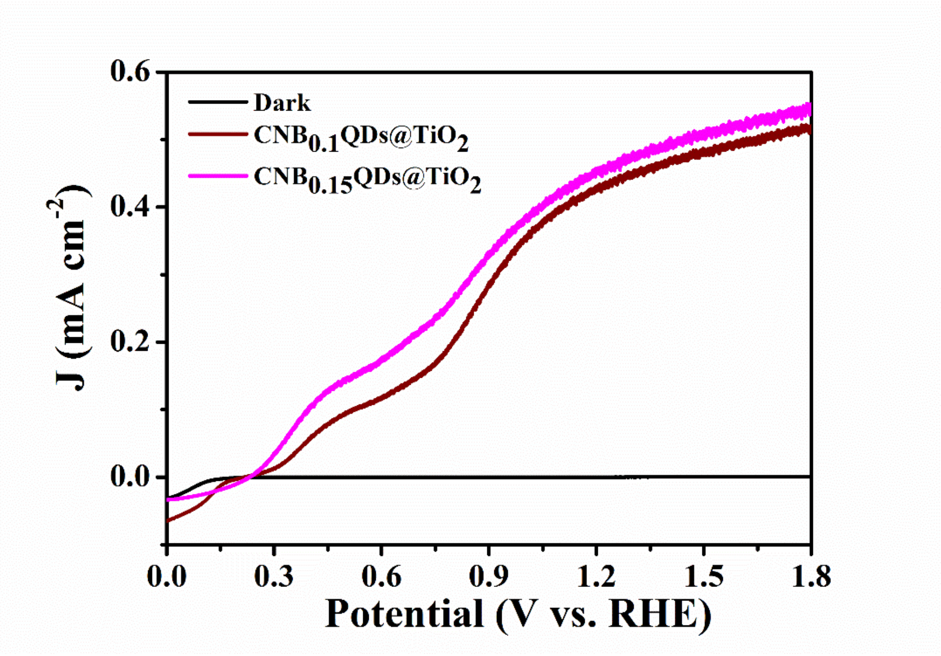

Figure S5. Linear sweep voltammograms of $CNB_{0.1}QDs$ @ TiO_2 and $CNB_{0.15}QDs$ @ TiO_2 composite.

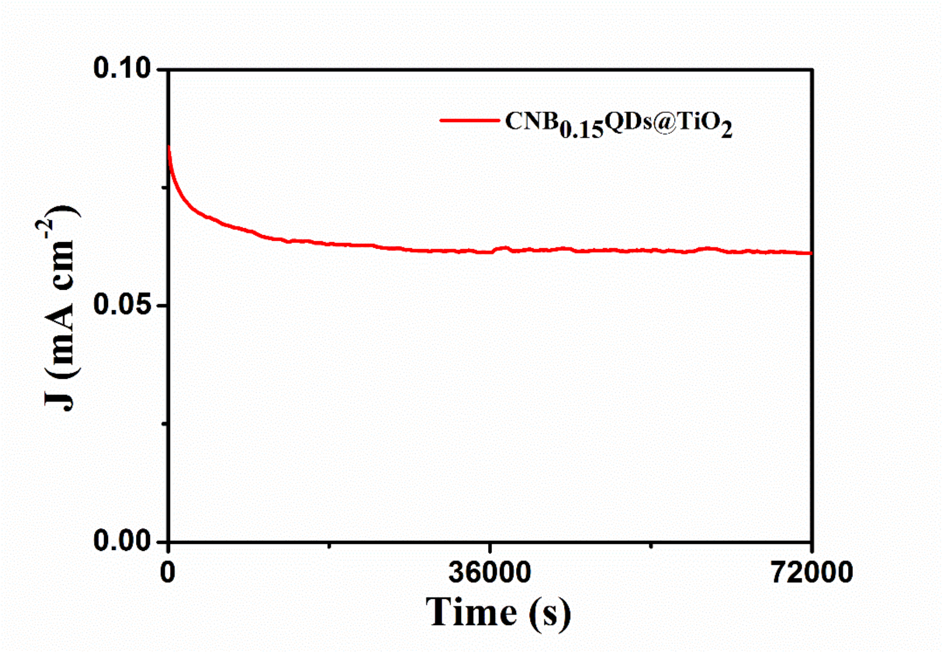

Figure S6. I-t curves of $CNB_{0.15}QDs@TiO_2$ sample at 0.65 V (vs RHE) for 72000 s (12 h) under continuous simulated sunlight illumination.
